# ﻿A new species of *Phyllophichthus* Gosline, 1951 (Actinopterygii, Ophichthidae) from Taiwan

**DOI:** 10.3897/zookeys.1220.126594

**Published:** 2024-12-09

**Authors:** Yusuke Hibino, Shunsuke Endo, Hsuan-Ching Ho

**Affiliations:** 1 Kitakyushu Museum of Natural History and Human History, Fukuoka 805-0071, Japan; 2 Fishery Research Laboratory, Kyushu University, Fukuoka 811-3304, Japan; 3 Center for Advanced Technical and Educational Supports, Faculty of Agriculture, Kyushu University, Fukuoka 819-0395, Japan; 4 Department and Graduate Institute of Aquaculture, National Kaohsiung University of Science and Technology, Kaohsiung 811, Taiwan; 5 Australian Museum, Sydney 2010, Australia

**Keywords:** Biodiversity, fish fauna, ichthyology, marine species, nasal tube, snake eel, taxonomy

## Abstract

A unique species of the flappy-snake eel genus, *Phyllophichthusdiandrus***sp. nov.**, is described based on a single specimen (270 mm in total length) collected from Dong-gang, southwestern Taiwan. The new species possesses several characters that are distinct from the only other species in the genus, *Phyllophichthusxenodontus*. *Phyllophichthusdiandrus***sp. nov.** can be easily distinguished from *P.xenodontus* by having two papillae inside of nasal tube (vs three in *P.xenodontus*), 25 branchiostegal rays (vs 29), the dorsal-fin origin positioned behind the tip of the pectoral fin (vs not behind, usually above mid-pectoral fin), and the absence of the maxillary teeth (vs present). The relationship between *Phyllophichthus* and *Leiuranus* is discussed based on generic and morphological features.

## ﻿Introduction

The family Ophichthidae (snake eels or worm eels) is the most abundant group of the order Anguilliformes, comprising 365 species in 62 genera. While many species are recognized, most of them are belonging to several major genera, i.e. *Apterichtus* Duméril, 1805 (20 spp.), *Bascanichthys* Jordan & Davis, 1891 (19 spp.), *Ophichthus* Ahl, 1789 (97 spp.), and *Scolecenchelys* Ogilby, 1897 (20 spp.) (Y. Hibino pers. data). In contrast, several specialized monotypic genera have been discovered, such as *Glenoglossa* McCosker, 1982, which has an elongate tongue with a shrimp-like tip, and *Chauligenion* McCosker & Okamoto, 2016, which has a protruding lower jaw.

The genus *Phyllophichthus* Gosline, 1951 is one of the unique monotypic genera, established for *Phyllophichthusxenodontus* Gosline, 1951. Gosline’s (1951) work, which included an osteological study, was based on various groups of Ophichthidae. He found specialization of the shape of the anterior nasal tube that has a leaf- or flower-like shaped, posterior extension in *Phyllophichthus* (Gosline 1951; Hibino and Kimura 2016; Hibino 2020). In addition, *Phyllophichthus* has an acute, straight snout, as well as relatively simple sensory canals on the head.

During a survey in recent decades by our team of the snake-eel fauna of Taiwan, one particular specimen was collected that was initially identified as *Phyllophichthus* sp. by Hibino (2019), who suggested the possibility of it being an undescribed species but also noted a potential abnormality in the lower jaw. After careful re-examination, we have determined it to be the second species within the genus.

## ﻿Materials and methods

All methods for morphological measurements follow Hibino et al. (2019). Measurements for total and tail lengths are taken by 300 or 600 mm rulers and others by a digital caliper to the nearest 0.1 mm. Osteological observation including counts of vertebrae and branchiostegal rays are based on the radiographs taken by a digital x-ray machine (Dexela CMOS X-ray detector, Model 2315, Dexeal Co. Ltd, UK) in the National Museum of Marine Biology and Aquarium, Pingtung, Taiwan. Total length (**TL**) and head length (**HL**) are used throughout for expressing the body proportions.

DNA extraction, polymerase chain reaction (PCR), and sequencing methods followed Weigt et al. (2012). The total DNA was extracted from muscle tissue of samples and amplified partial regions of mitochondrial genes of 16S ribosome RNA (16S) and cytochrome c oxidase subunit I (COI). The primers for PCR and sequence were primer A (5′-GGTCCWRCCTGCCCAGTGA-3′), B (5′-CCGGTCTGRACYAGATCACGT-3′) for 16S (Kurogi 2008) and FISH-BCL (5′-TCAACYAATCAYAAAGATATYGGCAC-3′), FISH-BCH (5′-ACTTCYGGGTGRCCRAARAATCA-3′) for COI (Baldwin et al. 2009). Amplification of the target region was confirmed by gel electrophoresis, DNA was purified by ExoSAP-IT (Applied Biosystems) and sequenced using 3730×l DNA Analyzer (Applied Biosystems). All sequences have been deposited in the DNA Data Bank of Japan (**DDBJ**) (Table 1).

**Table 1.** Accession numbers of sequences for the present study.

Species	Accession no.		Voucher collection no.	Locality
	16S	COI		
*Brachysonophiscirrocheilos*	LC815008	LC815014	FRLM 47057	Dong-gang, Taiwan
*Echelusuropterus*	LC815009	LC815015	FRLM 47022	Dong-gang, Taiwan
*Leiuranussemicinctus*	OP035206		USNM 446167	Wallis and Futuna
*Leiuranusversicolor*	LC506441	LC815016	KPM-NI 50816	Miyazaki, Japan
*Muraenichthyshattae*	LC599668	LC815025	OMNH-P 38345	Osaka, Japan
*Myrichthysmaculosus*	LC815010	LC815017	FRLM 38878	Wakayama, Japan
*Ophichthuscelebicus*	LC599662	LC815018	FRLM 49737	Ha Long Bay, Vietnam
*Ophichthuserabo*	LC599664	LC815019	KMNH VR 100283	Nha Trang, Vietnam
*Ophichthusurolophus*	LC815011	LC815020	FRLM 47025	Dong-gang, Taiwan
*Ophichthuszophistius*	LC599660	LC815021	FRLM 36624	Shima, Mie, Japan
*Phyllophichthusdiandrus* **sp. nov.**	LC815012	LC815022	NMMB-P28224	Dong-gang, Taiwan
*Phyllophichthusxenodontus*	LC815013	LC815023	KMNH VR 100268	Okinawa, Japan
*Scolecenchelysaoki*	LC599667	LC815024	FRLM 38979	Mie, Japan

Partial sequences of 16S (537 bp) and COI (612 bp) obtained present study and retrieved GenBank (Table 1) were aligned by MAFFT v. 7 (Katoh and Standley 2013) and a maximum-likelihood (ML) tree (16S + COI) was constructed by RAxML v. 8 (Stamatakis 2014). The evolutionary model was used GTR+G+I following the best model computing by MEGA 11 (Tamura et al. 2021). The confidence of each branch was evaluated by 1000 bootstrap replications. *Scolecenchelysaoki* (Jordan & Snyder, 1901) and *Muraenichthyshattae* Jordan & Snyder, 1901 were used as outgroups.

Specimens examined in this study are deposited in the Kitakyushu Museum of Natural History and Human History, Kitakyushu, Fukuoka, Japan (**KMNH VR**), National Museum of Marine Biology and Aquarium, Pingtung, Taiwan (**NMMB-P**), and Museum Support Center of the Smithsonian Institution, Suitland, Maryland, USA (**USNM**).

### ﻿ Phyllophichthus diandrus sp. nov.

Taxon classificationAnimaliaAnguilliformesOphichthidae

DC450565-2FD2-5BF0-BD47-2B8301CC1149

https://zoobank.org/7134B3C8-04EB-4E43-9761-1AC65F70A8BE

Figs 1 , 2A , 3 , 4

Phyllophichthus sp.: Hibino 2019: 149 (Dong-gang, Taiwan).

#### Type material.

***Holotype***: NMMB-P28224, 270 mm TL, ca 22°26'N, 120°24'E, Dong-gang, Pingtung, southwestern Taiwan, northern South China Sea, mid-water trawl, 6 Jan. 2017, collected by H.-C. Ho.

#### Diagnosis.

Inside of anterior-nostril tube with two papillae; dorsal-fin origin well behind tip of pectoral fin, distance from the tip to the origin of dorsal fin 0.6 times of the fin; no teeth on maxilla and vomer; 25 branchial aches; vertebral formula 12-69-160.

#### Description.

**Figure 1.**
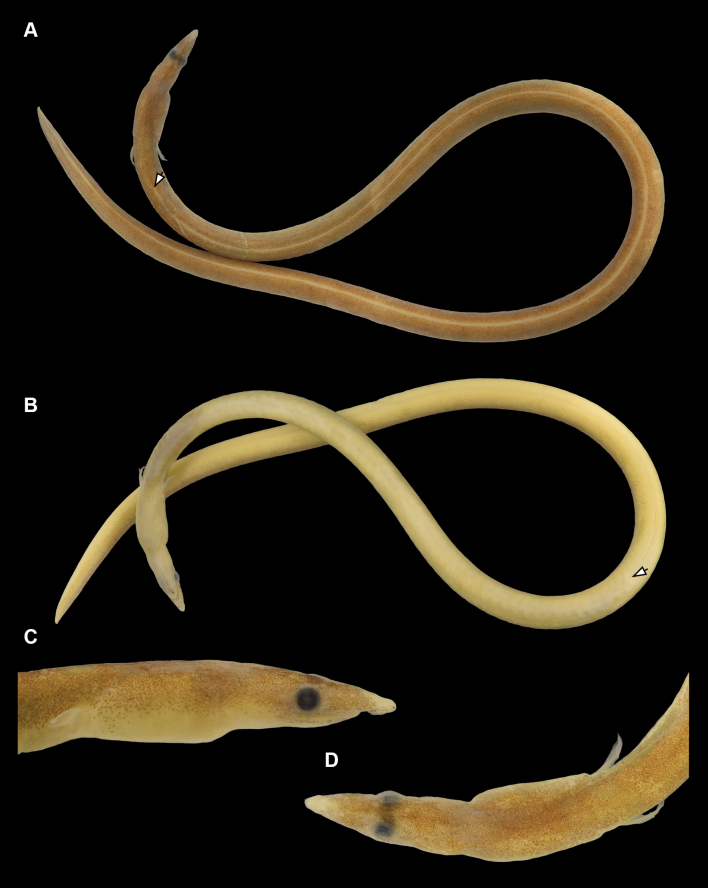
*Phyllophichthusdiandrus* sp. nov., NMMB-P28224, holotype, 270 mm TL, Dong-gang, Taiwan **A** dorsal view **B** ventral view **C** enlarged view of lateral head. Arrows indicate origin of dorsal fin (above) and anus (below).

Measurements in mm (% of TL in parenthesis): HL 22.4 (8.3%); preanal length 128.1 (47.5%); tail length 141.7 (52.5%); predorsal length 29.8 (11.0%); body depth at gill opening 4.7 (1.7%); body depth at mid anus 6.2 (2.3%). Measurements in mm (% of HL) in parenthesis: snout length 5.2 (23.2%); eye diameter 1.8 (8.0%); mouth gape 5.4 (24.1%); interorbital width 1.6 (7.1%); gill opening 1.9 (8.5%); isthmus 2.7 (12.1%); pectoral-fin length 3.2 (14.3%); pectoral-fin base 1.5 (6.7%). Body elongate, slender, trunk cylindrical, compressed toward tip of tail; tip of tail hard and pointed; anus anterior to mid-body, tail 1.9 in total length (Fig. 1).

**Figure 2.**
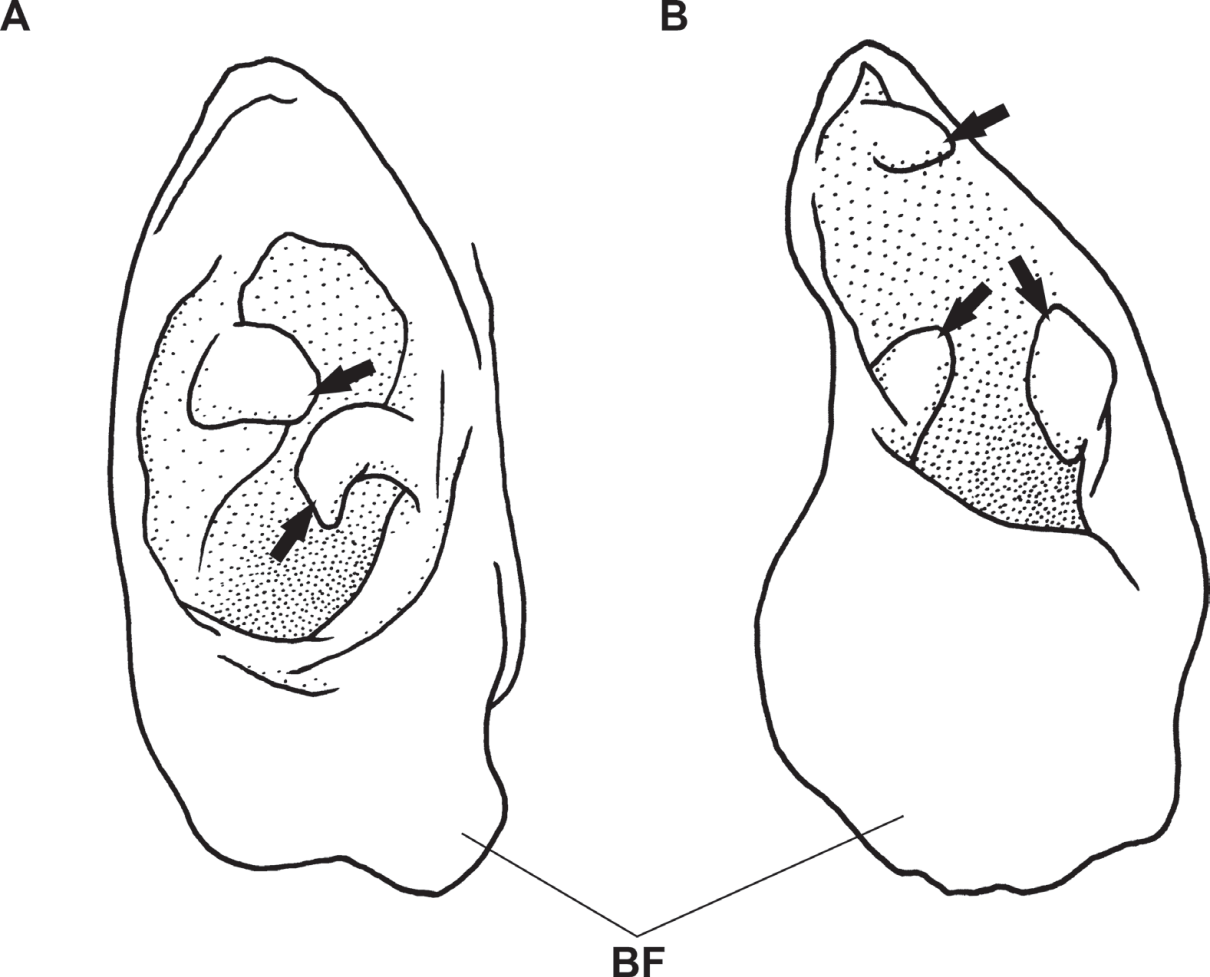
Closed illustration of tubular anterior nostril from ventral view **A***Phyllophichthusdiandrus* sp. nov., NMMB-P28224, holotype, 270 mm TL**B***P.xenodontus*, NMMB-P5264 (one of two), 373 mm TL, Taiwan. **BF** broad flap. Arrows indicate papillae inside of nasal tube.

Head relatively short, 5.7 in preanal length and 12.0 in TL; contour of head smooth, slightly convex in post-temporal; snout acute and relatively pointed in lateral view, narrow from dorsal side; snout long, prominently projected anteriorly; a distinct groove ventrally of snout; mouth inferior, lower jaw short and tip below middle of base of nostril tube, distance between tips of snout and lower jaw more than eye diameter; anterior nostril tubular, towards ventrally, base of both side closed: posterior rim of tube extending posteriorly, forming a broad flap; inside of tube with two papillae (Fig. 2A); posterior nostril a slit opening inside of mouth, concealed by wide dermal flap rolling into mouth completely; eye developed, large, its diameter 2.9 times in snout length; lips smooth without cirri or protrusions, upper lip extending to inner mouth, a curved fold along lower margin of eye; mouth moderate, rictus slightly behind a vertical through posterior margin of eye; interorbital region slightly convex without groove; branchial basket convex; gill opening positioned just anterior to base of pectoral fin.

**Figure 3.**
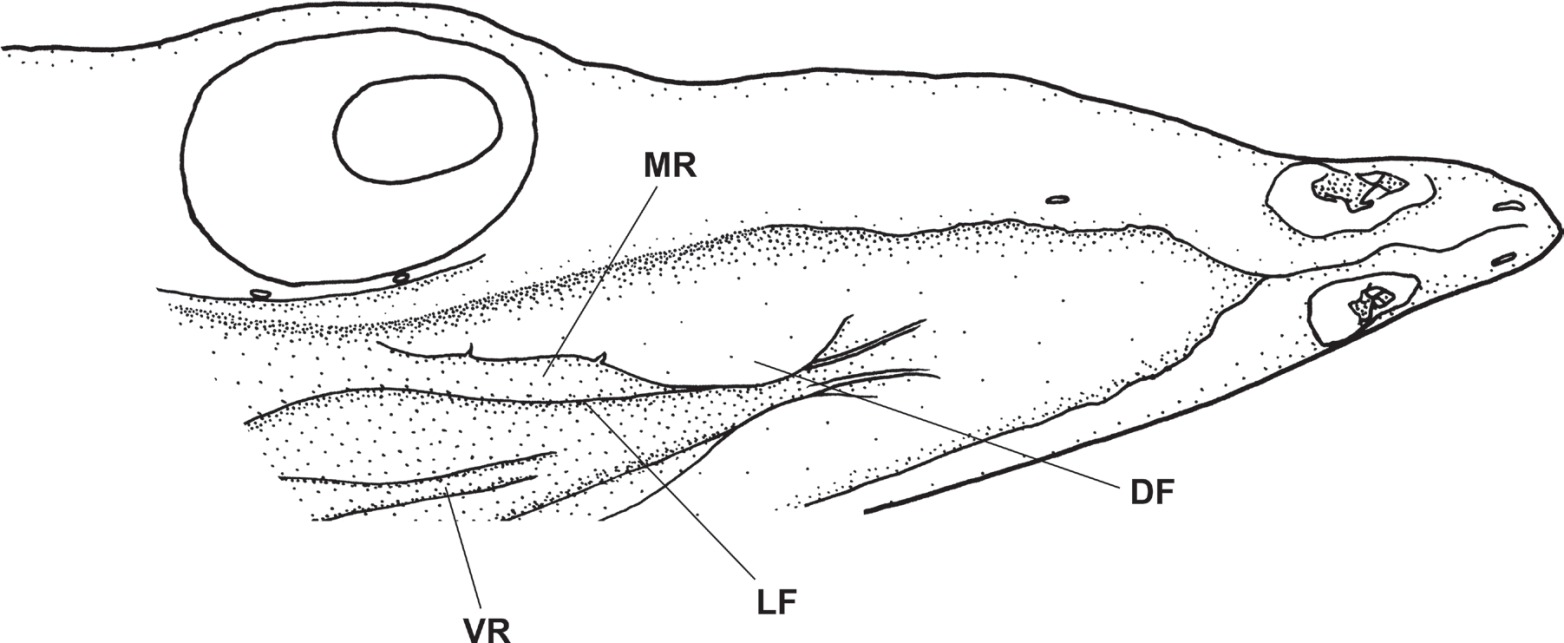
Ventrolateral view of palatal area of *Phyllophichthusdiandrus* sp. nov., NMMB-P28224, holotype, 270 mm TL. **DF** dermal flap on posterior nostril **LF** edge of upper labial flap **MR** maxillary region **VR** vomerine region.

Teeth small, conical, pointed; no teeth on maxilla and vomer, maxillary region completely covered by an extending upper labial flap (Fig. 3); 6 intermaxillary teeth arranged in two rows but concealed by a dermal structure and not visible; dentary teeth uniserial posteriorly, 17 on left side and 15 on right side.

**Figure 4.**
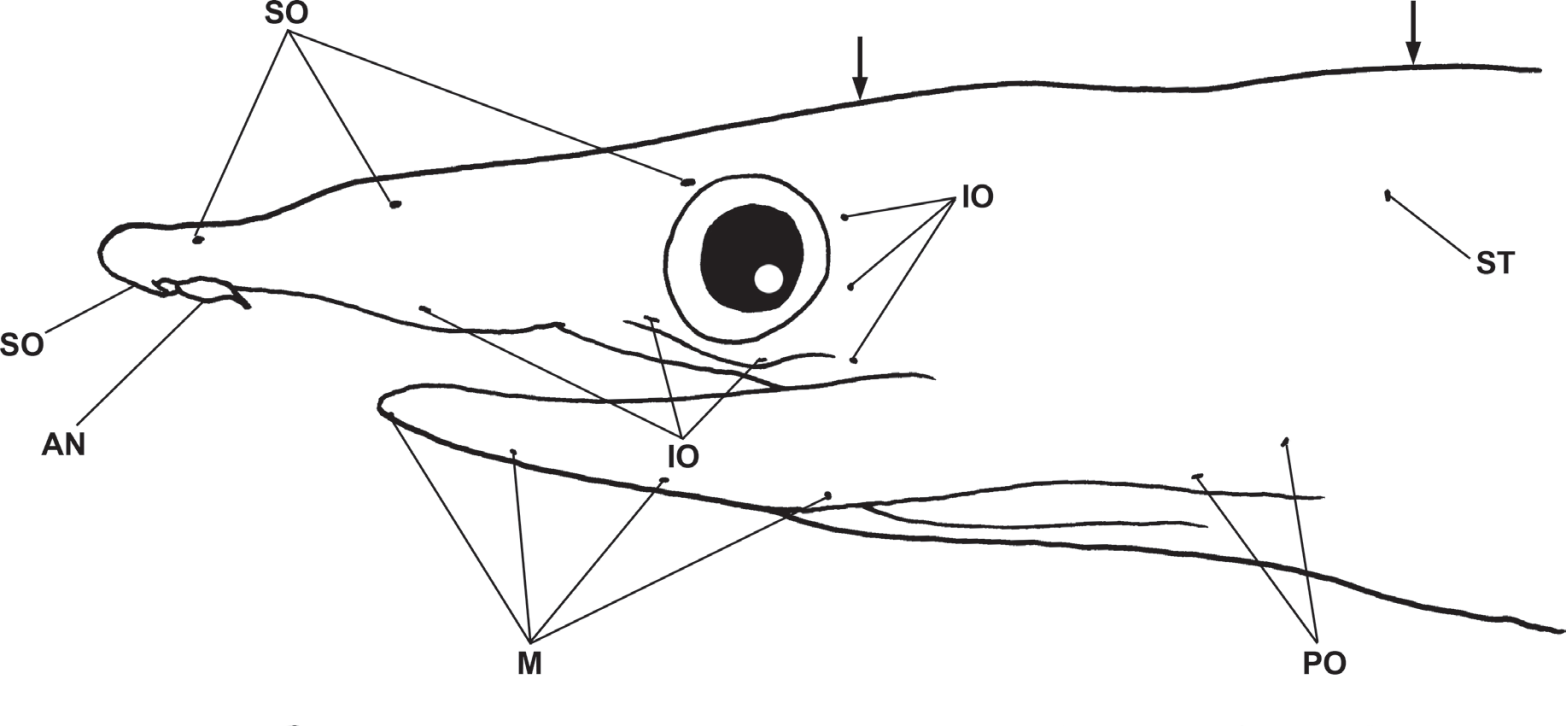
Lateral view of anterior head of *Phyllophichthusdiandrus* sp. nov., NMMB-P28224, holotype, 270 mm TL. **AN** anterior-nostril tube **IO** infraorbital pores **M** mandibular pores **PO** preopercular pores **SO** supraorbital pores **ST** supratemporal pore. Arrows indicate interorbital (left) and mid-temporal pores (right).

Sensory pores on head obvious (Fig. 4); supraorbital pores 1 + 3, 1 ethmoid and 3 pores slightly before a level of center of eye; infraorbital pores 3 + 3, first between anterior and posterior nostrils and second anteroventro-corner of eye, third behind center of eye, and remaining on postorbital; preoperculo-mandibular pores 4 + 2; 3 on supratemporal; 1 interorbital pore present; lateral-line pore slightly obscure, nearly completed except tip of tail equal to one third of HL.

Neurocranium narrow dorsally; dentary stout and recurved; branchiostegal rays developed and deeply overlapped ventrally, 25 in total. Predorsal vertebrae 12, preanal 69, and total 160.

Pectoral fin moderate, shape round, its length 1.6 in snout and 7.0 in HL; medial fins low in height, origin of dorsal fin behind tip of pectoral fin, distance from pectoral fin-tip to dorsal-fin origin 0.6 times of the fin, distance from gill opening to dorsal-fin origin 3.0 in HL; caudal fin absent.

Color in preservation light yellowish brown; abdomen slightly paler but not bicolored distinctly; all fins pale white.

#### Distribution.

Known only by a single specimen from Dong-gang, southwestern Taiwan. Depth range estimated as more than 100 m.

#### Etymology.

The specific name *diandrus* is the Latin compound adjective “bi-stamened”, referring to the flower-like shaped tubular nostril with two papillae inside.

## ﻿Discussion

Based on careful examination of the radiograph images of the holotype, we found no osteological damage on its lower jaw, despite the suspicion of an abnormal condition raised by Hibino (2019). As stated above, *Phyllophichthus* was known previously only from a single species, *P.xenodontus* which is widespread in the tropical and subtropical regions in the Indo-Pacific Ocean.

Our species represents the second species in the genus. *Phyllophichthusdiandrus* sp. nov. can be easily distinguished from *P.xenodontus* by having two papillae inside of nasal tube (vs three in *P.xenodontus*), 25 branchiostegal rays (vs 29), the dorsal-fin origin behind the tip of the pectoral fin (vs usually above mid-pectoral fin), and an absence of the maxillary teeth (vs present) (McCosker 1977; Smith et al. 2014; this study). Another nominal species, *Phyllophichthusmacrurus* McKay, 1970, described from Western Australia, has been regarded as a junior synonym of *P.xenodontus* by several authors (McCosker et al. 2006; Smith et al. 2014; McCosker 2022). The dorsal-fin origin of *P.macrurus* is positioned above posterior two-thirds of the pectoral fin, not behind the fin tip (McKay 1970; this study) and is clearly within the range of *P.xenodontus*. Thus, we retain this name in the synonymy of *P.xenodontus*.

Smith et al. (2014) noted that because of the wide range in the vertebral counts there might be several populations present in *P.xenodontus*. However, due to the small number of specimens, they did not separate them into different taxa. Smith et al. (2014) examined 11 specimens collected from Hawaii (type locality of *P.xenodontus*), Seychelles, the Red Sea, Taiwan, Solomon Islands, Vanuatu, and the Marquesas Islands. In their description, they clearly stated that *P.xenodontus* has three papillae inside the nasal tube, the same as the illustration of the holotype provided by Gosline (1951). Five of 11 specimens examined by Smith et al. (2014) were also checked and this character was confirmed by YH. Furthermore, our additional specimens of *P.xenodontus* from Taiwan (*n* = 3) and Japan (*n* = 2) also have characters identical to the holotype.

**Figure 5.**
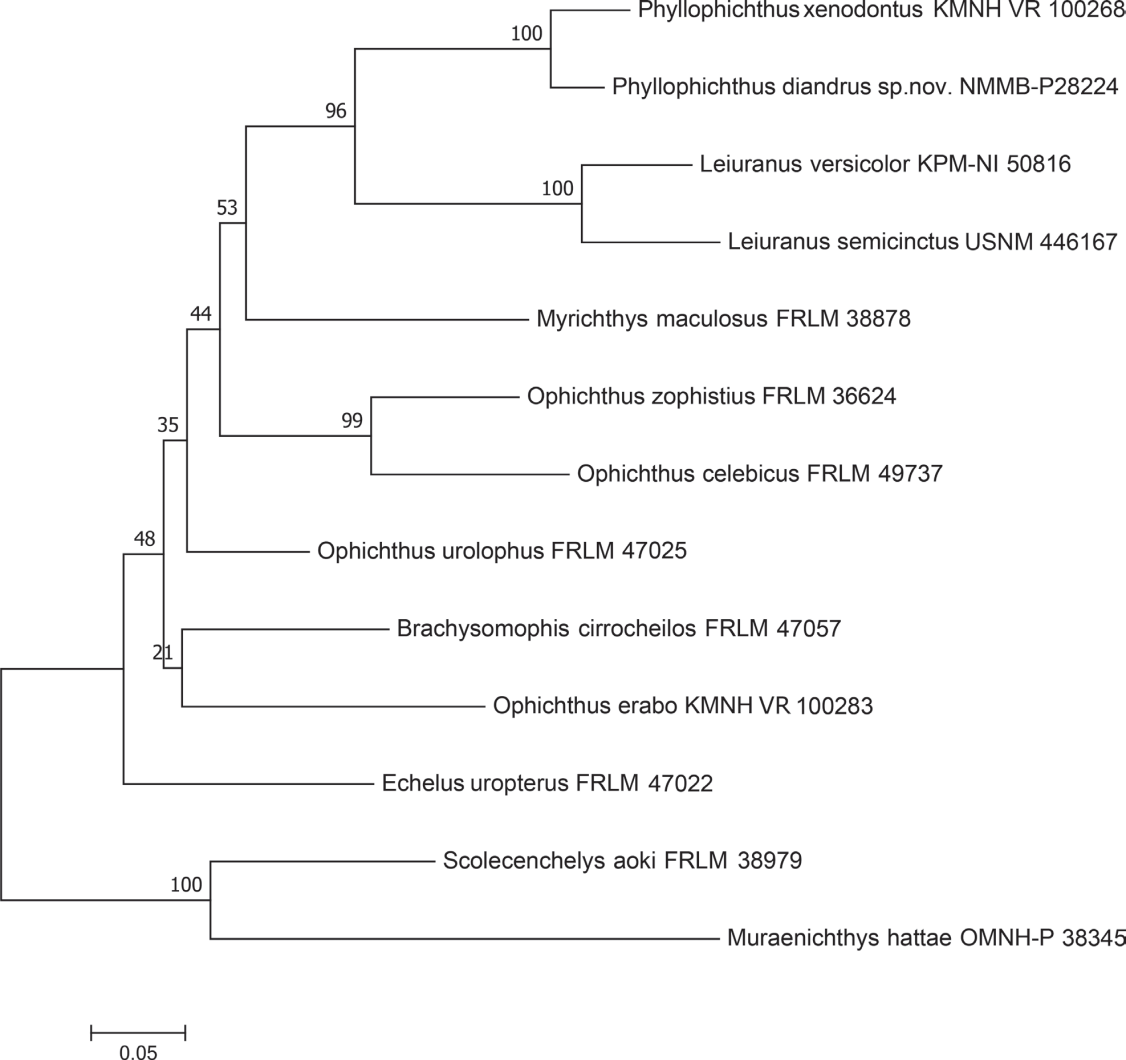
Maximum likelihood tree of selected genera in subfamily Ophichthinae based on partial COI and 16S sequences, including two *Phyllophichthus* species.

The genetic distance between *P.diandrus* sp. nov. and *P.xenodontus* is high, more than 5% based on a combination of mitochondrial COI and 16S sequences (Fig. 5). Furthermore, *P.diandrus* sp. nov. exhibits a distinctive characteristic of lacking teeth on the maxilla, a trait that may warrant consideration for the establishment of a new genus in Ophichthidae. However, further investigation with additional specimens and more comprehensive studies are necessary to explore this hypothesis.

The genus *Phyllophichthus* was established by Gosline (1951). While mentioning that its dentition is similar to *Leiuranus* Bleeker, 1852 (i.e. both genera lack vomerine teeth), he also noted that the condition of the mandibular teeth in *Phyllophichthus* is unique. McCosker (1977) confirmed *Phyllophichthus* as a valid genus and suggested that *Phyllophichthus* is closely related to *Leiuranus* and *Elapsopis* Kaup, 1856 in the tribe Ophichthini. McCosker et al. (1989) treated *Elapsopisversicolor* Richardson, 1848, the only species of *Elapsopis*, as a member of *Leiuranus* without further explanation. *Leiuranusversicolor* has vomerine teeth, free branchial rays, secondary ossification of the fifth ceratobranchial, and the presence of actinosts (McCosker 1977), but other osteological features, including fusion of the upper pharyngeal dermal tooth plates, are consistent with those of *Leiuranussemicinctus* (Lay & Bennett, 1839).

Based on COI and 16S sequences, *Phyllophichthus* forms a sister group with *Leiuranus*, supported by a high bootstrap value. This result supports the hypothesis proposed by McCosker (1977), which was primarily based on osteological analysis. While the shape of the neurocranium (subtruncate) of *Phyllophichthus* is similar to that of *Leiuranus*, the expanded appendage of the nasal tube, modified suspensorium, jaws, dentition, and neurocranium (McCosker 1977; this study) are distinct from the former. Consequently, we recognize both *Phyllophichthus* and *Leiuranus* as valid genera, each comprising two species.

Fricke et al. (2015) established a new genus and species, *Suculentophichthusnasus* Fricke, Golani & Appelbaum-Golani, 2015 from a single specimen collected at Eilat, Gulf of Aqaba in the Red Sea. Because of its large tubular nostril they considered the genus to be closely related to *Phyllophichthus*, distinguishing the two genera by a number of differences, including the shape of the tubular nostril, caudal vertebral counts, the position of the dorsal fin origin, and the presence of supraorbital canal/pores. However, the first three characters are within species-level variation, and there is no difference in the fourth character. It is notable that *Phyllophichthus* has no protrusions along the upper lip, whereas *S.nasus* has at least one protrusion (one in the text, but two shown in the illustration). Further work is needed to determine whether *Phyllophichthus* and *Suculentophichthus* Fricke, Golani & Appelbaum-Golani, 2015 should be regarded as separatable genera or not.

*Phyllophichthusdiandrus* sp. nov. is unique in having no teeth on the maxilla, which is completely covered by an extending upper labial flap. It is speculated that this species specializes in feeding on soft organisms due to the presence of dentary and intermaxillary teeth.

### ﻿Comparative materials

*Phyllophichthusxenodontus*: USNM 162709, holotype, 238 mm TL, Oahu Island, Hawaii Islands; WAM P.4015-001, holotype of *P.macrurus*, 465 mm TL, near Albany, Western Australia (photo examination only); KMNH VR 100268, 249 mm TL, USNM 132819, 269 mm TL, Okinawa Island, Ryukyu Islands, Japan; NMMB-P2753, 283 mm TL, NMMB-P5264, two specimens, 373–422 mm TL, Hsiao-liu-chiu Island, southwestern Taiwan; USNM 121374, 349 mm TL, Batan Island, Philippines; USNM 224232, 319 mm TL, Caroline Islands, Micronesia; USNM 314690, 204 mm TL, Aldabra Atoll, Seychelles; USNM 363698, 345 mm TL, Bank Islands, Vanuatu.

## Supplementary Material

XML Treatment for Phyllophichthus diandrus

## ﻿Additional information

### Author ORCIDs

Yusuke Hibino https://orcid.org/0000-0002-5670-3851

Hsuan-Ching Ho https://orcid.org/0000-0003-1154-601X

### Data availability

All of the data that support the findings of this study are available in the main text.
